# Outcome and Complications of Colonoscopy: A Prospective Multicenter Study in Northern Israel

**DOI:** 10.1155/2012/612542

**Published:** 2012-06-19

**Authors:** A. Suissa, O. S. Bentur, J. Lachter, K. Yassin, I. Chermesh, I. Gralnek, A. Karban, I. Khamaysi, Y. Naveh, A. Tamir, A. Shahbari, R. Eliakim

**Affiliations:** ^1^Rappaport Faculty of Medicine, Technion Israel Institute of Technology, Haifa, Israel; ^2^Departments of Gastroenterology and Community Medicine, Rambam Health Care Campus, Haifa, Israel; ^3^Department of Gastroenterology, Elisha Hospital, Haifa, Israel; ^4^Department of Gastroenterology, HaEmek Medical Center, Afula, Israel

## Abstract

*Background*. Colonoscopy for screening the population at an average risk of colorectal cancer (CRC) is recommended by many leading gastrointestinal associations. *Objectives*. The objective was to assess the quality, complications and acceptance rate of colonoscopy by patients. *Methods*. We prospectively gathered data from colonoscopies which were performed between October 2003 and September 2006. Patients were asked to return a follow-up form seven days after the procedure. Those who failed to do so were contacted by phone. *Results*. 6584 patients were included (50.4% males). The average age of subjects was 57.73 (SD 15.22). CRC screening was the main indication in 12.8%. Cecal intubation was achieved in 92% of patients and bowel preparation was good to excellent in 76.2%. The immediate outcome after colonoscopy was good in 99.4%. Perforations occurred in 3 cases—1 in every 2200 colonoscopies. Significant bleeding occurred in 3 cases (treated conservatively). 94.2% of patients agreed to undergo repeat colonoscopy in the future if indicated. *Conclusions*. The good quality of examinations, coupled with the low risk for complications and the good acceptance by the patients, encourages us to recommend colonoscopy as a primary screening test for CRC in Israel.

## 1. Background

In recent decades, colonoscopy has been established worldwide as the “gold standard” common procedure for the evaluation of the colon. Indications for colonoscopy include examining symptomatic individuals, performing followup on patients with colonic diseases and screening of healthy individuals for CRC. 

After lung cancer, CRC is the second most common cause of cancer-related death in the United States [1]. In Israel, CRC is the second most common malignancy in men and women with an annual occurrence of about 3000 new patients per year [2]. The precancerous lesion, the adenomatous polyp, progresses to CRC over a period of 4 to 12 years, therefore there is a golden opportunity for early detection as well as primary prevention by removing polyps. 5-year survival rates in CRC patients diagnosed early are over 90%, whereas they drop to under 10% when disease is diagnosed in advanced stages. Hence, CRC fulfills the criteria of a disease in which screening is highly efficient: high occurrence, long latent period, and an improved prognosis with early detection [3, 4]. An additional efficiency criterion fulfilled by this disease is the availability of efficient and cost-effective tests.

The American Gastroenterological Association, The American College of Gastroenterology, and the American Society for Gastrointestinal Endoscopy all recommend that people with an average risk for CRC should have one of several screening tests. Of the available screening methods, they recommend colonoscopy as the gold standard for average-risk and high-risk patients [1, 5, 6]. The average-risk group includes any person over the age of 50 (due to the dramatic increase in CRC incidence after this age) and the high-risk group includes people with a personal or family history of CRC/polyps, or one of the known syndromes such as Lynch (hereditary nonpolyposis CRC) or familial adenomatous polyposis. During recent years, colonoscopy has become the main method for early detection of CRC in various parts of the world.

The major complications of colonoscopy are colonic perforation or massive gastrointestinal bleeding. The reported rate of perforation in diagnostic and therapeutic colonoscopies ranges between 1/500 and 1/3000. The reported rate of major bleeding, which is more common when a polypectomy or biopsy is performed, is around 1/500 [7–10]. Retrospective studies performed in Israel have reported perforation rates of 1/1724 to 1/1358 [11, 12].

Common minor complications of colonoscopy include bloating, abdominal pain, mild bleeding, diarrhea, constipation, and nausea. These are usually mild and self-limited.

An additional annoying aspect of colonoscopy is the preparation for the examination. In one study, this was reported by 77% of patients as the most unpleasant part of the procedure. 

In Israel, the Ministry of Health has accepted the American gastrointestinal associations' recommendation to perform colonoscopy in patients with a high risk of CRC. However, screening colonoscopy is not included in the state-funded medical insurance for patients with an average risk of CRC. These are only offered testing for fecal occult blood.

The goals of the present study were to assess the quality, complications, and acceptance rates of colonoscopy by patients, in order to assess whether this test should be a routine examination for CRC screening in Israel.

## 2. Subjects and Methods

### 2.1. Study Population

**Figure 1:**
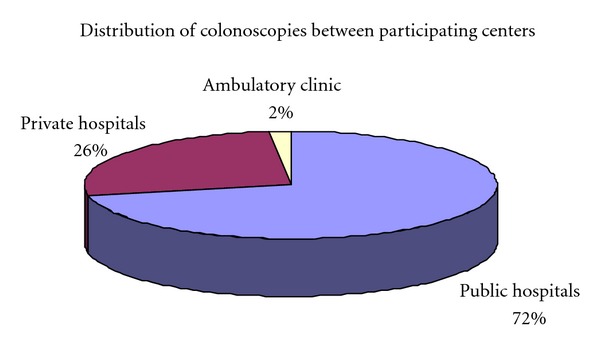


During the study period, between 09/2003 and 10/2006, data were gathered prospectively on inpatient and outpatient colonoscopies performed in 10 public or private hospitals and public out-patient clinics in northern Israel (see Figure 1). 

### 2.2. Study Protocol

At the end of each colonoscopy, the physician endoscopist filled out a form with parameters related to the procedure: demographic data, in-patient/out-patient procedure type, body constitution, prior surgery, indications for colonoscopy, premedication, bowel preparation, procedure duration, depth of insertion, invasive procedures (biopsy and polypectomy), immediate outcomes, and immediate complications. The patients were sent home with a questionnaire which they were asked to fill out and return (prepaid postage) 7–10 days after the procedure. This questionnaire included questions related to late or minor complications: pain, rectal bleeding, fever, or any complication mandating an emergency room visit. Patients were also asked to score how unpleasant the procedure was from 1 to 10. A high score (10) indicates minimal inconvenience. They were also asked if they would be willing to undergo an additional colonoscopy in the future.

The questionnaires were translated to the 3 common native languages in the region: Hebrew, Arabic, and Russian. Patients who failed to return the questionnaire were contacted by phone, and in every case of complication, the patients were prospectively followed until resolution. Some patients who failed to return the questionnaire were not reachable by phone. A sample of this group of patients was followed via a computerized database which contains hospitalization and death data regarding most of the population in Israel (OFEK: the integrated hospital-community electronic medical record). The study protocol was approved in advance by the institutional review board.

### 2.3. Statistical Analysis

Data were analyzed using SPSS 14.0. Descriptive statistical tests were used to calculate mean values, frequencies, and percentiles. Pearson's chi-square, Mann-Whitney and exact tests were used to compare frequencies. Independent sample *t*-tests and ANOVA were used to compare means.

## 3. Results

6584 colonoscopies were included in this study. 90.6% (5940) of the colonoscopies were out-patient procedures. 9.4% (617) were performed during hospitalization. 4 centers, including 2 public hospitals, an endoscopic unit in a private hospital and an ambulatory public clinic, actively participated in this study. 6 additional centers initially recruited very few patients and dropped out.

The average age of the subjects examined was 57.7 ± 15.2. 51.8% (3348) were over 50 years old. 

The colonoscopies were performed for the common clinical indications. The most common indications (see Table 1) for colonoscopy were rectal bleeding, changes in bowel habits, abdominal pain, and followup after a previous polypectomy. 12.8% (839) of colonoscopies were performed for screening purposes of individuals with either an average risk for CRC or a family history of the disease.

Midazolam was the most common type of premedication and was administered to 98.4% (6478) of patients (average dose 5 ± 1.7 mg). 2.1% (138) of patients received doses over 10 mg. Fentanyl was used in 88.3% (5812) of patients (average dose  102 ± 21  mcg), Pethidine in 5.2% (341) of patients (doses of 25–50 mg), and Propofol in 8.4%. Two examinations were performed under general anaesthesia.

Bowel preparation for colonoscopy was graded as good or excellent in 76.2% (4855) of examinations.

The duration of the examination from the insertion to the removal of the scope was <30 minutes in 94.2% (6169) of cases and <15 minutes in 53.9% (3527). A complete intubation of the cecum, terminal ileum, or anastomosis was achieved in 92% (5899). Biopsy and/or polypectomy were performed in 45.7% (2999) of examinations.

The immediate outcome was uneventful in 99.4% (6483) of patients. Adverse immediate events included 13 cases of severe abdominal pain, 10 cases of milder yet significant pain, 3 cases of major bleeding, and a single case of perforation. All cases of major bleeding were treated medically/endoscopically and occurred in patients who underwent either biopsy or polypectomy. The perforation was diagnosed immediately after colonoscopy. It occurred in an 82-year-old woman who came for polyp followup, no biopsy or polypectomy were performed during the procedure. She was successfully treated surgically. Two additional cases of perforation presented at later stages. One occurred in an 80-year-old man who had a polyp removed during CRC followup. The second perforation occurred in a young female with inflammatory bowel disease, in which multiple biopsies were taken. Both cases were treated conservatively. Thus, three cases of perforation occurred in the 6584 colonoscopies in this study, that is, a perforation rate of 1 in every 2194 examinations.

The patient questionnaires in this study were retrieved by two different methods: 59.8% were returned voluntarily by patients via mail or fax. 40.2% of the questionnaires were filled out over the phone. The patient questionnaires were retrieved by one of the two methods in 63.1% (4155) of cases. At least 50% of patient questionnaires were retrieved in each of the participating centers. 81.6% of patients reported an uneventful course, without any pain, substantial bleeding, fever, or emergency room visits related to the procedure. 14.1% reported substantial bleeding, 23 patients reported fever over 38°centigrade, and 22 reported visiting emergency medical services because of one of these minor complaints. Further analysis revealed that 64.6% of the patients who reported bleeding had been originally sent to colonoscopy for either rectal bleeding or inflammatory bowel disease.

The examination unpleasantness was graded by the patients from 1 to 10, high scores indicating a pleasant examination. The average score was 8.3 ± 1.7, the median score was 9.

94.2% of patients were willing to undergo an additional colonoscopy in the future if clinically indicated. This rate was 91.4% in the voluntarily returned questionnaires and 98.4% in those questionnaires retrieved by phone. The rate of pain reported in patient questionnaires was higher in voluntarily returned forms than in those retrieved by phone.

### 3.1. Screening Colonoscopies

12.8% (839) of the colonoscopies in this study were screening colonoscopies of patients with an average or increased risk of CRC. Bowel preparation, mean examination duration, and cecal intubation were significantly better in the screening colonoscopies than in colonoscopies for other indications. Screening colonoscopies had significantly less biopsies and polypectomies done than colonoscopies performed for other indications. The rates of biopsies and polypectomies in screening colonoscopies were 17% and 32.1%, respectively. Immediate outcome was uneventful in 99.6% of screening colonoscopies. The reported cases of perforation or major bleeding in our study did not occur in patients who had screening colonoscopy. The patient questionnaire was completed by 65% (545) of patients in the screening group. The rates of pain and blood in feces during the week following the examination were significantly lower compared to colonoscopies performed for other indications. 95.9% (521) of the screening group expressed willingness to undergo a repeat colonoscopy in the future (if clinically indicated) as compared to 94% of patients undergoing colonoscopy for nonscreening indications; however, this difference was not statistically significant.

### 3.2. Comparison between Different Centers

In accordance with the medical system structure in Israel, the colonoscopies in this study were performed in public hospitals, a public ambulatory clinic and an endoscopy unit of a private hospital. 86.9% of the procedures in the public hospitals were out-patient procedures, as were all the procedures in the clinic and the private hospital. Analysis showed no gross differences between the three types of centers. Of the slight differences noted, bowel preparation was better in the private hospital and the out-patient clinic (*P* < 0.01). A relatively low rate of polypectomies and biopsies, 30.4% was reported in the public clinic, in comparison to 46% in the public and private hospitals. Willingness to undergo additional colonoscopy was lowest in the public clinic, 85%, compared to 94% to 95% in the public and private hospitals.

## 4. Discussion

The primary objective of this study was to measure the main outcome parameters related to colonoscopy performance in Israel. This is the first large-scale prospective study on the outcome and complications of colonoscopies done in Israel.

Our data were collected prospectively from 6584 examinations performed for various indications.

Only 12.8% of examinations were labeled officially as screening tests. Since screening average-risk population by colonoscopy is not covered by the Israeli medical insurance, patients are sometimes sent by their primary care physician with an indication other than screening, for the test to be covered by insurance. For instance, 20% of our study population officially presented to colonoscopy because of rectal bleeding. Some of these patients might have had symptoms highly suggestive of hemorrhoidal disease, but their physicians chose to send them to colonoscopy with the indication of “rectal bleeding.” Others (15%) were sent with the indication of “abdominal Pain.” When taking into account that 75% of the subjects in our study population were over 50 years old, these two categories may add to a significant percentage of screening colonoscopies done.

The willingness to respond to the questionnaires was over 60%. Since patient questionnaires were collected in two different ways, we assessed their validity. Indeed, we found some differences between voluntarily returned questionnaires and those filled out over the phone after we contacted the patients, with mainly more minor complications and less satisfaction in the voluntary group. The questionnaires which were filled over the phone were obviously filled out at a later date than those returned voluntarily, and this finding might suggest that patients tend to forget the minor complications of colonoscopy with time and that as time passes, they might become more willing to undergo an additional colonoscopy. This finding is undoubtedly encouraging to the efforts to install an effective CRC screening program, in which repetition of colonoscopy every 5–10 years is crucial for success.

The results of our study show that colonoscopies are performed in Israel in a safe and effective manner. The effectiveness of colonoscopy is measured by parameters such as bowel preparation, the rate of cecal intubation, and the rate of biopsies and polypectomies performed during colonoscopy. 76.2% of patients had good-to-excellent bowel preparation, the rate of cecal intubation was 92%, compared to 93–99% reported in the literature [12, 13]. 23.7% of examinations included polypectomies, compared to 23.5–42% reported in the literature [12, 14, 15]. Safety parameters included perforation and major bleeding rates. There were no fatalities reported in our study. The rate of perforations (1/2200) is comparable to the rates of 1/500–1/3000 reported in the literature. All these are a testament to the efficiency and safety of the procedure in Israel.

Patients expressed a high acceptance (94.2%) to undergo repeat colonoscopy in the future. This is a fundamental prerequisite for establishing an effective CRC screening program, since screening colonoscopies should be repeated after 10 years in an average-risk population and sooner in other cases. Patients undergoing colonoscopies with an indication of primary screening expressed an even higher willingness to undergo additional colonoscopies in the future (95.9%). The better positive feedback in this group may be attributed to their higher awareness of the importance of the procedure. After all, these are patients who willingly came to have a colonoscopy and in some instances even paid for the examination, since it was not officially covered by the state medical insurance.

Presuming screening colonoscopy for average-risk population will be funded by medical insurance in Israel, the examinations will most probably be performed in various medical centers. For this reason, our study included public and private hospitals and a public out-patient clinic. We compared the quality of colonoscopy between the different centers. We did not find any substantial or consistent differences between the various centers performing colonoscopy.

Our study measured various colonoscopy-related parameters. One parameter which must be addressed is premedication dosage, specifically Midazolam. 2.1% (138) of patients received doses higher than 10 mg, above the common recommended dosage, which can lead to severe side effects, including respiratory depression. These results suggest taking more caution with premedication and the preprocedural evaluation whether an anesthesiologist is needed.

Our results suggest that colonoscopy in Israel is a reliable, good-quality examination, with a low risk for complications and good acceptance by the patients. All these justify our recommendation that colonoscopy can be adopted as the primary screening test for CRC in Israel.

## Figures and Tables

**Table 1:** Indications for colonoscopy.

	Numbers (%)
Rectal bleeding	21.9% (1,432)
Changes in bowel habits	15% (981)
Polyp followup	13.5% (884)
CRC followup	6.3% (412)
Positive FOBT	3.7% (243)
Primary screening	5.6% (368)
Family history of CRC	7.2% (471)
Abdominal pain	15% (985)
Abnormal imaging	3.1% (205)
Anemia	5.9% (388)
IBD followup	3.1% (204)
Other	6.7% (440)

*The study protocol allowed for a maximum of 3 indications for each patient.
